# The Fluoroquinolone Finafloxacin Protects BALB/c Mice Against an Intranasal Infection With *Francisella tularensis* Strain SchuS4

**DOI:** 10.3389/fmicb.2019.00904

**Published:** 2019-05-01

**Authors:** Kay B. Barnes, Karleigh A. Hamblin, Mark I. Richards, Thomas R. Laws, Andreas Vente, Helen S. Atkins, Sarah V. Harding

**Affiliations:** ^1^Defence Science and Technology Laboratory, Salisbury, United Kingdom; ^2^MerLion Pharmaceuticals, Berlin, Germany; ^3^University of Exeter, Exeter, United Kingdom; ^4^London School of Hygiene & Tropical Medicine, London, United Kingdom

**Keywords:** finafloxacin, *Francisella tularensis*, therapeutic, *in vivo* efficacy, biothreat

## Abstract

The efficacy of the novel fluoroquinolone finafloxacin was evaluated as a potential therapeutic *in vitro* and *in vivo*, following an intranasal infection of *Francisella tularensis* strain SchuS4 in BALB/c mice. We demonstrated that short treatment courses of finafloxacin provide high levels of protection, with a single dose resulting in a significant increase in time to death when compared to ciprofloxacin. In addition, following investigation into the window of opportunity for treatment, we have shown that finafloxacin can provided protection when administered up to 96 h post-challenge. This is particularly encouraging since mice displayed severe signs of disease at this time point. In summary, finafloxacin may be a promising therapy for use in the event of exposure to *F. tularensis*, perhaps enabling the treatment regimen to be shortened or if therapy is delayed. The efficacy of finafloxacin against other biological threat agents also warrants investigation.

## Introduction

*Francisella tularensis* is a highly infectious Gram negative bacterium that is the causative agent of the disease tularemia. There are two main clinically relevant sub-species, Type A (*F. tularensis* subspecies *tularensis*) is the most virulent, causing the most severe infections in humans. The second sub-species, Type B (*F. tularensis* subspecies *holartica*) is typically associated with less severe disease (Narayanan et al., 2018). *Francisella* can be transmitted by a number of routes including via bites by infected arthropods, ingestion of contaminated food and water or by inhalation. Due to its high infectivity (less than 10 organisms are required to establish infection in humans by the inhalational route of exposure), *F. tularensis* is considered to be a potential biothreat agent and has been classified as a category A Agent and a Tier 1 select agent (Dennis et al., 2001; Griffin et al., 2007; Roberts et al., 2018).

There is currently no licensed tularemia vaccine available. Historically, the Live Vaccine Strain (LVS) of *F. tularensis* was used routinely to vaccinate humans, however, the level of protection offered by LVS and the limited characterization with regards to its attenuation and protective response resulted in problems with licensure (Ellis et al., 2002). The mortality rate following exposure of *F. tularensis* by the inhalational route is up to 60% if left untreated, reducing to 2% with medical intervention (Evans et al., 1985; Gill and Cunha, 1997). The currently approved treatments for severe tularemia are the aminoglycosides (typically gentamicin), the tetracyclines and chloramphenicol (Kingry and Petersen, 2014). Although the aminoglycosides are recommended and very effective, they are administered parenterally which would not be practicable in the event of a large biological release. In addition, these drugs are associated with severe toxicity, and as such, alternative therapies are required. For mild to moderate cases of tularemia, the fluoroquinolones, particularly ciprofloxacin and levofloxacin, or a tetracycline (typically doxycycline) are considered to be further options (Sutera et al., 2014). Delay in initiation of treatment or administration of an inappropriate treatment can result in relapse of disease, reported in patients receiving ciprofloxacin or doxycycline (Chocarro et al., 2000; Dennis et al., 2001; Pérez-Castrillón et al., 2001; Boisset et al., 2014). The outcome is more likely to be positive if the duration between the development of symptoms and the initiation of therapy is short (Hepburn and Simpson, 2008).

Finafloxacin is a novel fluoroquinolone that has enhanced *in vitro* activity in acidic conditions where other fluoroquinolones are typically less effective (Smith et al., 1988; Stubbings et al., 2011). In addition, finafloxacin has good *in vitro* activity at neutral pH. This improved activity at low pH has been demonstrated against a number of Gram negative and Gram positive organisms including *Acinetobacter baumannii* and *Staphylococcus aureus* (Higgins et al., 2010; Lemaire et al., 2011). The use of finafloxacin would have obvious advantages in the treatment of infections caused by bacteria that can invade, survive and replicate in an intracellular environment, where the pH is low (Lemaire et al., 2011). A topical formulation of finafloxacin has been recently approved for the treatment of acute otitis externa in the United States (McKeage, 2015). It is also available in oral and intravenously delivered formulations, and is currently being developed for the treatment of urinary tract infections in hospitalized patients. Phase 2 trials have demonstrated that 800 mg of finafloxacin, administered for 5 days is as efficient as a 10 day regimen and that 800 mg of finafloxacin administered for 5 days is more efficient than the standard treatment (10 days of ciprofloxacin) (Wagenlehner et al., 2018). Efficacy in these trials was determined according to guidelines published by the FDA regarding the development of drugs for the treatment of complicated urinary tract infections (FDA, 2018 guidelines). Recently, we have shown that the oral formulation of finafloxacin has utility in treating infection with the biothreat agent *Burkholderia pseudomallei* in a BALB/c mouse model of melioidosis (Barnes et al., 2017). It has been suggested that this superior efficacy at infection-relevant pH (in addition to its activity at neutral pH) is due to ability to maintain activity and not be affected by eﬄux (Randall et al., 2017).

Given that *F. tularensis* invades multiple cell types and replicates intracellularly (thus protecting itself from the host immune response and therapeutic intervention), we considered it possible that finafloxacin may offer an advantage over currently recommended treatments by entering infected host cells and remaining active within the acidic environment of the phagosome where *F. tularensis* is known to briefly reside before moving into the cytosol (Bosio, 2011; Ozanic et al., 2015). We hypothesize that finafloxacin may offer improved efficacy in comparison to other fluoroquinolones (e.g., ciprofloxacin), that have reduced activity at a low pH, both in the early stage of infection (when *F. tularensis* is located in acidic environments such as the phagosome) and later (when it has escaped into conditions at neutral pH, e.g., the cytosol). The activity of ciprofloxacin has been shown to reduce when the pH is lowered (Akova et al., 1999). This antibiotic is very effective at bacterial killing in neutral conditions but is limited in acidic conditions, which for bacterial species like *F. tularensis* may result in dissemination through the host.

This work investigates the *in vitro* activity of finafloxacin against *F. tularensis* strains SchuS4 (Type A) and HN63 (Type B) and the *in vivo* efficacy of finafloxacin in a BALB/c model of inhalational tularemia.

## Materials and Methods

### Bacteria

For the *in vitro* assays, 10 μl (containing approximately 3.4 × 10^8^ CFU) of a frozen stock of *F. tularensis* strain SchuS4 (Larsson et al., 2005) or HN63 (Thomas et al., 2003) was added to 10 ml of Modified Cysteine Partial Hydrolysate (MCPH) broth, supplemented with cysteine (100 μg/ml) and glucose (4%), (Eyles et al., 2008) and incubated at 37°C, with shaking at 180 rpm for 24 h. The culture was then adjusted to an optical density of 0.1 at 590_nm_ in MCPH broth to obtain an inoculum of approximately 10^8^ CFU/ml. Further dilutions in MCPH broth were made according to the assay used. The growth of both *F. tularensis* strains was investigated at pHs 5–7. 0.1 M hydrochloric acid was used to adjust the pH of the broth which was checked prior to use.

For the *in vivo* studies, 10 μl (containing approximately 3.4 × 10^8^ CFU) of a frozen stock of *F. tularensis* strain SchuS4 was added to 10 ml of phosphate buffered saline (PBS.) This was adjusted to an optical density of 0.1 at 590_nm_.to obtain an inoculum of approximately 10^8^ CFU/ml. A serial dilution was performed in PBS to obtain the required challenge dose of 100 CFU. These dilutions were also plated onto blood cysteine glucose agar (BCGA) (Ireland et al., 2011) and incubated at 37°C for 48 h to calculate the actual dose delivered. Samples harvested from animals were plated onto BCGA + LCAT selective supplement (lincomycin, colistin sulfate, amphotericin B, trimethoprim), (Thermo Fisher Scientific) to prevent growth of commensal bacteria. All bacteriological procedures were carried out in a Class III microbiological safety cabinet or a Class III half suit rigid isolator within an Advisory Committee on Dangerous Pathogens (ACDP) Containment Level 3 laboratory.

### Animals

The animal studies were carried out in accordance with the United Kingdom Animals (Scientific Procedures) Act 1986 and the codes of practice for the Housing and Care of Animals used in Scientific Procedures 1989. Female BALB/c mice (Charles River Laboratories, United Kingdom) aged 8–10 weeks were randomized into cages of 5 within a Class III half suit rigid isolator in an ACDP Level 3 laboratory. Mice had free access to water and rodent diet (Harlan Teklad, United Kingdom) and underwent a 5–7 day acclimatization period before any procedures were performed.

### Antibiotics

Finafloxacin was supplied by MerLion Pharmaceuticals Pte Ltd. For the *in vitro* assays, 100 mg of finafloxacin or ciprofloxacin (Sigma Aldrich Ltd., United Kingdom) was added to 9 ml of sterile water and 1 ml of 1 M sodium hydroxide to make a working stock of 10 mg/ml. The equivalent concentration of sodium hydroxide, used to prepare the antibiotics, was included as a control for growth of bacteria. This control was included in all *in vitro* assays.

For the *in vivo* studies, a 15 mg/ml solution of finafloxacin was prepared by adding 2.1 ml of 0.01 M Tris buffer to 44 mg of finafloxacin (containing 37.5 mg of active ingredient). 200 μl of 1 M sodium hydroxide was added to dissolve the antibiotic, followed by 200 μl of 0.01 M hydrochloric acid. The pH of the resulting solution was pH 8. An intravenous preparation of ciprofloxacin (Ciproxin 2 mg/ml) was purchased from Bayer (Basingstoke, United Kingdom). Dosing regimens were determined using pharmacokinetic data generated from previous studies (Barnes et al., 2017; Hamblin et al., 2017).

### Minimum Inhibitory Concentrations (MICs)

The MICs for finafloxacin and ciprofloxacin were determined for *F. tularensis* strains SchuS4 and HN63 using the broth micro dilution method in accordance with the Clinical and Laboratory Standards Institute (CLSI) guidelines (with the exception of the bacterial growth conditions as *F. tularensis* did not grow in Mueller Hinton media) (CLSI, 2015). Assays were performed in 96 well plates in MCPH broth supplemented with cysteine (100 μg/ml) and glucose (4%) with antibiotic concentrations in the range of 64 μg/ml to 0.004 μg/ml, and using bacteria at a final concentration of approximately 5 × 10^6^ CFU/ml. Plates were incubated at 37°C for 48 h. This was performed in media at pH 7. Each assay was performed in triplicate and repeated three times. The MIC was recorded as the lowest concentration of antibiotic which prevented growth of the bacteria in the inoculum.

### Minimum Bactericidal Concentrations (MBCs)

The MBCs for finafloxacin and ciprofloxacin were determined by plating 100 μl aliquots of the MIC dilutions showing no visible growth onto BCGA plates in triplicate and incubating at 37°C for 72 h. The MBC was recorded as the lowest concentration of antibiotic that killed 99.9% of the bacteria in the inoculum. The assays were performed in accordance with the CLSI guidelines (with the exception of the bacterial growth conditions) (CLSI, 2015).

### Time Kill Assays

Time kill assays were performed at 4 × MIC, in accordance with the CLSI guidelines (with the exception of the bacterial growth conditions) (CLSI, 1999). Antibiotic solutions of finafloxacin and ciprofloxacin were prepared in 10 ml of MCPH broth supplemented with cysteine and glucose (as above) and adjusted to pH 6 or pH 7. Broths were inoculated with *F. tularensis* SchuS4 or HN63 at a concentration of approximately 5 × 10^6^ CFU/ml. Bacteria grown in the absence of antibiotic were also included. All broths were incubated shaking at 180 rpm at 37°C. Samples were taken at 0, 2, 4, 6, and 24 h, and a 10 fold serial dilution was performed in sterile PBS, plated onto BCGA and incubated at 37°C. Bacteria were enumerated after incubation for 72 h. A bactericidal effect was defined as a 3 log_10_ reduction or greater in CFU/ml compared with the original inoculum; a bacteriostatic effect was defined as up to a 3 log_10_ reduction in CFU/ml compared with the original inoculum. All samples were taken in duplicate and each assay was performed in triplicate.

### *In vivo* Efficacy Studies

Groups of mice were anesthetized with isoflurane (Isocare, Animal Care Limited, United Kingdom) and, once sedated, 50 μl of *F. tularensis* strain SchuS4 (prepared as described above) was delivered to the nares via a micropipette. In the first study, therapy was initiated at 24 h post-challenge. Groups of ten mice were dosed with 50 μl finafloxacin (37.5 mg/kg) orally, via pipette every 8 h, or with 300 μl ciprofloxacin (30 mg/kg) by the intraperitoneal (IP) route every 12 h. Control groups of infected mice were dosed with 50 μl of diluent (consisting of Tris Buffer, sodium hydroxide, and hydrochloric acid, adjusted to pH 8) orally, via pipette every 8 h, or with 300 μl of PBS via the IP route every 12 h. One group of ten mice were infected and untreated. Antibiotic treatment was continued for 1, 3, or 7 days.

Mice were observed twice daily for clinical signs of disease until day 33 post-challenge when the experiment was terminated. Upon reaching a cumulative, clinical score of 4 (demonstrating overt clinical signs of infection including ruﬄing and hunching) in the untreated animals, 5 mice receiving finafloxacin or ciprofloxacin for 1 day or 3 days, the oral diluent or IP administered PBS, were culled. Post-mortems were performed and the spleen, liver and lungs were harvested, weighed and homogenized into 1 ml of PBS. A 10 fold serial dilution was performed and 100 μl aliquots were plated onto BCGA + LCAT plates. Following incubation at 37°C for 72 h, the bacteria were enumerated, to determine the bacterial load in the organs. At day 33, all surviving mice were culled and the spleen, liver and lungs harvested and processed as above.

In the second study, mice were anesthetized and infected with *F. tularensis* strain SchuS4 via the intranasal route as detailed above. Therapy was initiated at 24, 48, 72, or 96 h post-challenge. Groups of 10 mice received antibiotics and control solutions at doses detailed in the first study. Again, one group of 10 mice was infected and untreated. Treatment regimens continued for 7 days. Mice were observed twice daily, for clinical signs of disease and weighed once daily for 35 days. At day 35, all surviving mice were culled and the spleen, liver and lungs were harvested, weighed and the bacterial load determined.

### Statistical Analysis

Some analyses have been performed using the program SPSS V21.0 (IBM) and some analyses and the graphs prepared using PRISM v6.0 (Graphpad). The time kill data were analyzed using two-way ANOVA with Tukey’s multiple comparison tests. Log Rank (Mantel-Haenszel) was used to analyze survival. Animal body weights were compared using a repeated measures General Linear Model (GLM) with Bonferroni’s post-tests. Mood’s Median test with pairwise adjustment was used to analyze bacterial burden. Organ weights were compared using Mann–Whitney *U*-tests and Dunn’s multiple comparisons and the clinical scores compared using the Kruskall Wallis test and Dunn’s multiple comparisons. Differences of *p* < 0.05 were considered statistically significant.

## Results

### Growth of *F. tularensis* at Different pHs

Growth of *F. tularensis* could not be initiated in media adjusted to pH 5. Therefore, the MICs for both *F. tularensis* SchuS4 and HN63 were performed at pH 7 only. *F. tularensis* strain SchuS4 (but not HN63) also grew in a 10 ml volume in media adjusted to pH 6, therefore time kill studies were performed at pH 6 and pH 7 for *F. tularensis* SchuS4 only. To induce sufficient growth, an inoculum of 5 × 10^6^ CFU/ml was required.

### MICs and MBCs

MIC’s and MBC assays were performed to determine the concentration of antibiotic required to inhibit or kill bacteria, respectively. In all assays, media containing sodium hydroxide at the same concentration used to dissolve the antibiotics was included as a control. The MIC for finafloxacin and ciprofloxacin were performed in supplemented MCPH at pH 7 for both *F. tularensis* SchuS4 and HN63. Finafloxacin demonstrated comparable activity to ciprofloxacin against both strains of *F. tularensis* [MICs of 0.016–0.03 μg/ml compared to 0.03 μg/ml for ciprofloxacin (Table 1)]. In MBC assays, finafloxacin demonstrated improved killing (with MBCs of 0.25–0.5 μg/ml) compared to ciprofloxacin (1 μg/ml) (Table 1).

**Table 1:** The MICs and MBCs of finafloxacin and ciprofloxacin for *F. tularensis* strains SchuS4 and HN63.

	MIC (μg/ml)		MBC (μg/ml)	
	Finafloxacin	Ciprofloxacin	Finafloxacin	Ciprofloxacin
***F. tularensis* SchuS4**	0.016	0.03	0.5	1
***F. tularensis* HN63**	0.03	0.03	0.25	1

*MIC assays were performed in supplemented MCPH broth adjusted to pH 7. MBCs were determined by plating aliquots of the MIC dilutions showing no visible growth onto BCGA plates.*

### Time Kill Assays

**FIGURE 1:**
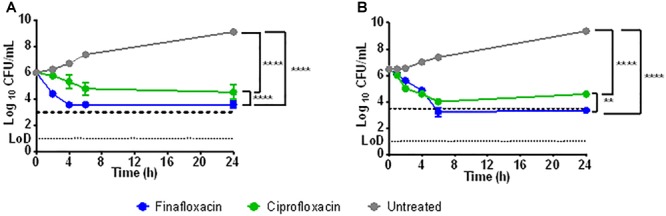
The activity of finafloxacin and ciprofloxacin against *F. tularensis* strain SchuS4 over 24 h. Time kill assays were performed in supplemented MCPH broth adjusted to pH 6 **(A)** or pH 7 **(B)**, at 4 × MIC of finafloxacin (0.064 mg/L) or ciprofloxacin (0.12 μg/ml). An untreated bacterial culture was included. The dotted line represents a 3 log_10_ reduction in CFU/ml from the starting inoculum. Statistics was performed using a two-way ANOVA with Tukey’s multiple comparison test. ^∗∗^*p* < 0.01, ^∗∗∗∗^*p* < 0.0001. Each data point is the mean of 3 replicates and the error bars represent the standard error. LOD, limit of detection.

Time kill assays were performed to investigate how rapidly the antibiotics had an effect on the viability of *F. tularensis* strain SchuS4. Finafloxacin or ciprofloxacin at 4 × MIC (0.064 μg/ml and 0.12 μg/ml for finafloxacin and ciprofloxacin, respectively) were added to supplemented MCPH broth adjusted to pH 6 or pH 7. They were incubated with *F. tularensis* and sampled over a 24 h period. At pH 6, both finafloxacin and ciprofloxacin were bacteriostatic; however, incubation with finafloxacin resulted in a 2.5 log_10_ reduction in CFU/ml during the first 4 h of the assay, unlike ciprofloxacin which remained bacteriostatic during this period (Figure 1). At pH 7, finafloxacin demonstrated bactericidal activity over a 24 h period compared to ciprofloxacin which had a bacteriostatic effect (Figure 1). Both antibiotics had a significant effect when compared to the untreated control at both pHs (*p* < 0.0001). Finafloxacin had superior activity to ciprofloxacin at both pH 6 (*p* < 0.0001) and pH 7 (*p* < 0.01) when the time kill curves were compared in totality.

### *In vivo* Efficacy Studies

**FIGURE 2:**
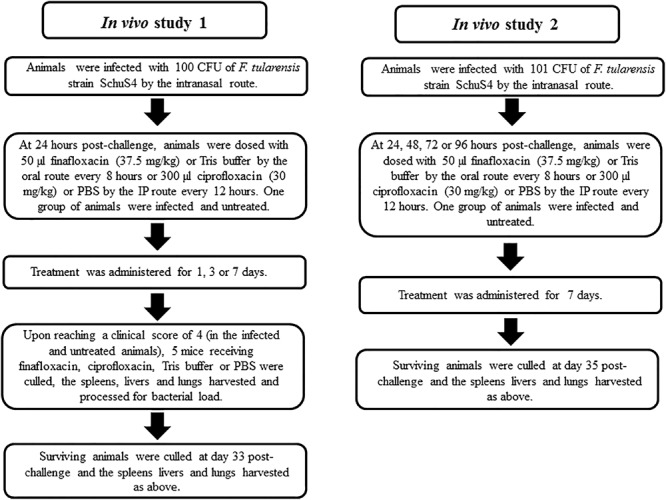
A graphical schema detailing the two *in vivo* studies discussed in the manuscript.

**FIGURE 3:**
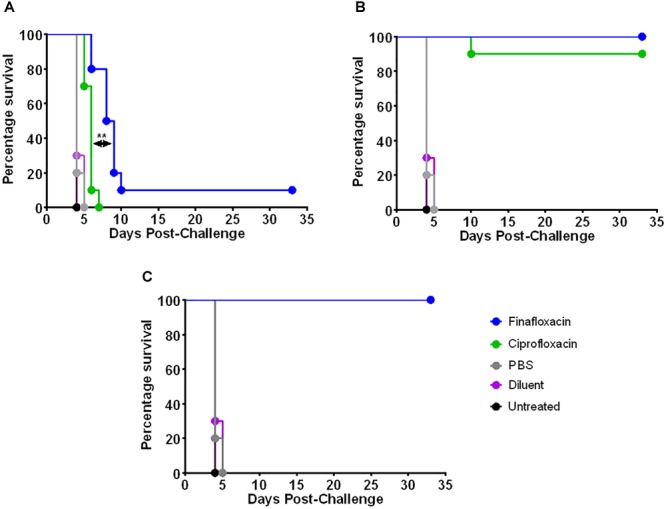
The percentage survival of mice following challenge with *F. tularensis* SchuS4 by the intranasal route. Mice were challenged with 100 CFU of *F. tularensis* by the intranasal route and treated with finafloxacin (37.5 mg/kg) by the oral route every 8 h or ciprofloxacin (30 mg/kg) by the intraperitoneal route every 12 h. Regimens were initiated at 24 h post-challenge, and continued for 1 **(A)**, 3 **(B)**, or 7 **(C)** days. Control animals received PBS by the IP route or diluent by the oral route. A group of animals were infected and received no treatment. Statistical analysis was performed using a Mantel-Haenszel log rank test. ^∗∗^*p* < 0.01.

The two *in vivo* efficacy studies are detailed in Figure 2. The first study investigated the protection offered by different durations of antibiotic treatment. Animals were infected with approximately 100 CFU of *F. tularensis* strain SchuS4 by the intranasal route, equating to approximately 10 median lethal doses. Mice were dosed from 24 h post-challenge with a human equivalent dose of finafloxacin or ciprofloxacin. All untreated mice and those that received the control substances (diluent or PBS) succumbed to infection by day 5 post-challenge. There was no significant difference in survival between these control groups (*p* > 0.05) (Figure 3). Both antibiotics offered an improved level of protection than either control substance (*p* < 0.001). All of the mice that received 1 day of ciprofloxacin treatment succumbed to infection by day 7 post-challenge (Figure 3A). Mice treated with finafloxacin also succumbed but demonstrated a significant increase in time to death when compared to ciprofloxacin (*p* < 0.01). Nine mice treated with finafloxacin succumbed to infection by day 10 post-challenge with one mouse surviving until the end of the study (Figure 3A). Survival at the end of the study was 100 and 90% for mice that received 3 days of finafloxacin and ciprofloxacin treatment, respectively; with no significant difference in survival offered by the two antibiotics (*p* > 0.05) (Figure 3B). Similarly, both finafloxacin and ciprofloxacin offered 100% protection when 7 days of treatment was delivered (Figure 3C).

**FIGURE 4:**
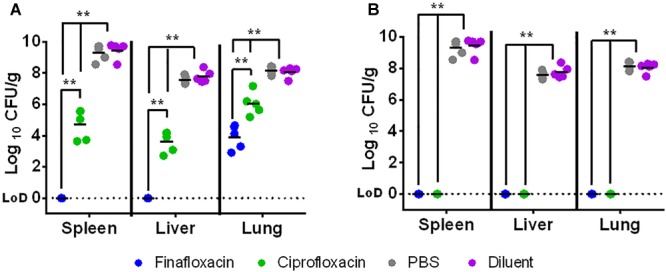
The bacterial load in organs at day 4 following challenge with *F. tularensis* SchuS4 by the intranasal route. Mice were challenged with 100 CFU of *F. tularensis* by the intranasal route and treated with finafloxacin (37.5 mg/kg) by the oral route every 8 h or ciprofloxacin (30 mg/kg) by the intraperitoneal route every 12 h until the control animals reached an average clinical score of 4 when 5 mice per group were culled and their organs harvested. Therapy was initiated at 24 h and continued for 1 day **(A)** or 3 days **(B)**. Control animals received PBS by the IP route or diluent by the oral route. Statistical analysis was performed using pairwise comparisons of a Mood’s Median test. ^∗∗^*p* < 0.01. The lines within the individual data points are the means of the log_10_ transformed data for each group.

When the untreated animals reached an average clinical signs score of 4 (at day 4 post-challenge, demonstrating overt clinical signs of infection including ruﬄing and hunching), 5 mice from the groups receiving 1 or 3 days of finafloxacin or ciprofloxacin (or control substances) were culled and their spleens, livers and lungs were harvested for bacterial enumeration. There was a significant reduction in the bacterial load within the organs of animals receiving 1 day of finafloxacin or ciprofloxacin treatment (Figure 4A) when compared to the control animals (*p* < 0.01). There were no bacteria detected in the spleen or liver of mice that received finafloxacin. In comparison, *F. tularensis* was recovered from all 3 organs harvested from mice treated with ciprofloxacin, and the bacterial load was significantly greater than in those organs harvested from mice treated with finafloxacin (*p* < 0.01). Collectively, we observed a reduced level of bacteria present in all organs of mice treated with 1 day of finafloxacin when compared to ciprofloxacin.

No bacteria were recovered from the spleens, livers or lungs of mice treated with finafloxacin or ciprofloxacin for 3 days (and culled at day 4 post-challenge) (Figure 4B) compared to the control animals which had bacterial loads of 2.06 × 10^7^–5.84 × 10^9^ CFU/ml (*p* < 0.01). At day 33 post-challenge, the study was terminated, all of the surviving mice were culled and their spleens, livers and lungs were harvested for bacterial enumeration. No *F. tularensis* was recovered from the organs harvested from animals treated with finafloxacin or ciprofloxacin.

**FIGURE 5:**
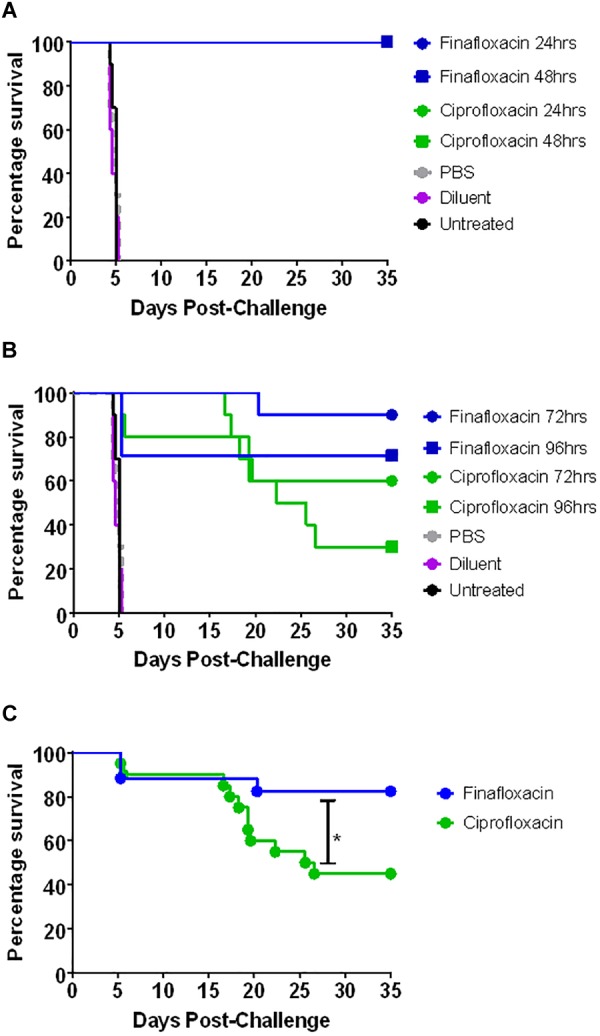
The percentage survival of mice following challenge with *F. tularensis* SchuS4 by the intranasal route. Mice were challenged with 101 CFU of *F. tularensis* by the intranasal route and treated with finafloxacin (37.5 mg/kg) by the oral route every 8 h or ciprofloxacin (30 mg/kg) by the intraperitoneal route every 12 h. Regimens were initiated at 24 or 48 h **(A)** or 72 or 96 h **(B)** post-challenge, and continued for 7 days. Control animals received PBS by the IP route or diluent by the oral route. A group of animals were infected and received no treatment. Survival data for the groups receiving therapy at 72 and 96 h have been combined **(C)**. Statistical analysis was performed using the Mantel-Haenszel log rank test. ^∗^*p* < 0.05.

The second *in vivo* study investigated the window of opportunity for delivering antibiotic therapy and investigated additional outputs including differences in weight profile and clinical score. The intranasal dose of *F. tularensis* strain SchuS4 received by the mice was calculated as approximately 101 CFU. All untreated mice and those that received the control substances succumbed to infection by day 5 post-challenge (Figure 5). There was no significant difference in survival between the three control groups (untreated and vehicle control treated animals) (*p* > 0.05). Treatment with finafloxacin and ciprofloxacin significantly improved survival when compared to the untreated controls and those receiving the control substances (*p* < 0.001). All mice receiving finafloxacin or ciprofloxacin initiated at 24 or 48 h post-challenge for 7 days survived the infection with *F. tularensis* (Figure 5A). At 72 h post-challenge, mice did not display any overt signs of disease; however, following 7 days of therapy initiated at 72 h, one mouse treated with finafloxacin and four mice treated with ciprofloxacin succumbed to infection (Figure 5B). This difference in the level of protection offered by ciprofloxacin and finafloxacin at the end of the study was not significant (*p* > 0.05).

At 96 h post-challenge mice displayed severe signs of infection (ruﬄing, hunching, eye discharge impairing vision, and rapid breathing). Three mice from the group scheduled to be dosed with finafloxacin at this time were excluded from the study as they reached their humane endpoint before therapy was initiated. Following therapy, a further two mice treated with finafloxacin succumbed to infection with *F. tularensis* (Figure 5B). Three mice that received ciprofloxacin initiated at 96 h post-challenge survived until the end of the study, compared to five mice treated with finafloxacin. There was no significant difference in survival between mice receiving the two therapies at this time point (Figure 5B). Stratified analysis of the 72 and 96 h data (the groups where some mortality was observed) combined together demonstrated a benefit of treating with finafloxacin compared to ciprofloxacin (*p* < 0.05) (Figure 5C).

**FIGURE 6:**
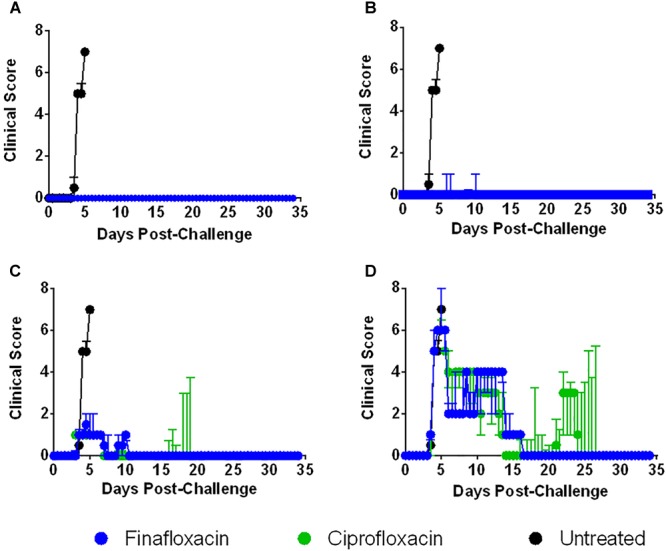
The clinical scores of mice infected with *F. tularensis* and treated with finafloxacin and ciprofloxacin. Mice were challenged with 101 CFU of *F. tularensis* by the intranasal route and treated with finafloxacin (37.5 mg/kg) by the oral route every 8 h or ciprofloxacin (30 mg/kg) by the intraperitoneal route every 12 h. A group of animals were infected and received no treatment. Mice receiving the control substances displayed very similar patterns of clinical signs to the untreated control group (data not included in the Figure). The graphs show the median daily summed clinical scores per treatment group when antibiotics were initiated at 24 **(A)**, 48 **(B)**, 72 **(C)**, or 96 h **(D)** post-challenge and continued for 7 days. The error bars are the interquartile ranges.

Infecting BALB/c mice with *F. tularensis* by the intranasal route resulted in the development of severe clinical signs of disease. When treatment with finafloxacin or ciprofloxacin was initiated at 24 or 48 h post-challenge, mice developed mild, transient clinical signs of disease (Figure 6A,B). Delaying the initiation of therapy to 72 h post-challenge also had a considerable benefit, the severity of clinical signs of disease were significantly reduced when compared to the untreated controls (*p* < 0.01) (Figure 6C). As described above, at 96 h post-challenge, mice displayed severe clinical signs; however, administering finafloxacin and ciprofloxacin even at this late stage did produce a beneficial response. Those mice that survived 48 h following therapy initiation largely resolved their clinical signs and only displayed ruﬄing (Figure 6D). The data may also indicate that more mice treated with ciprofloxacin relapsed with infection upon cessation of therapy, compared to those treated with finafloxacin (developed clinical signs of disease following the resolution of the initial infection) (Figure 6D), although larger group sizes would be required to confirm this suggestion. Statistical analysis may be biased as several mice treated at 72 and 96 h post-challenge succumbed to infection before relapse, therefore the data generated is from mice with a higher tolerance to the infection.

**FIGURE 7:**
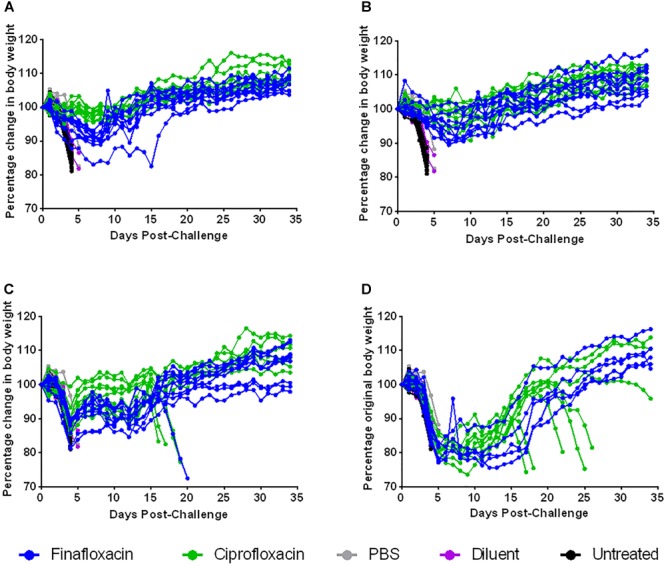
The percentage weight change following ciprofloxacin and finafloxacin therapy in mice challenged with *F. tularensis* SchuS4 by the intranasal route. Mice were challenged with 101 CFU of *F. tularensis* by the intranasal route and treated with finafloxacin (37.5 mg/kg) by the oral route every 8 h or ciprofloxacin (30 mg/kg) by the intraperitoneal route every 12 h. Control animals received PBS by the IP route or diluent by the oral route. A group of animals were infected and received no treatment. The graphs show the percentage change in weight over time when antibiotics were initiated at 24 **(A)**, 48 **(B)**, 72 **(C)**, or 96 h **(D)** post-challenge and continued for 7 days. Each line represents an individual mouse.

Animal weights were also recorded daily throughout this study. When therapy was initiated at 24 or 72 h post-challenge, treatment with ciprofloxacin resulted in a reduction in weight loss compared to treatment with finafloxacin (*p* < 0.01 up to day 35 when therapy was initiated at 24 h and *p* < 0.01 up to day 16 when therapy was initiated at 72 h) (Figure 7A,C). There were no differences in weight loss when therapy was initiated at 48 or 96 h post-challenge, although the data recorded for the 96 h groups could only be analyzed up to day 5 as animals started to succumb to infection at this point in the study, thus leaving incomplete groups (Figure 7B,D).

**FIGURE 8:**
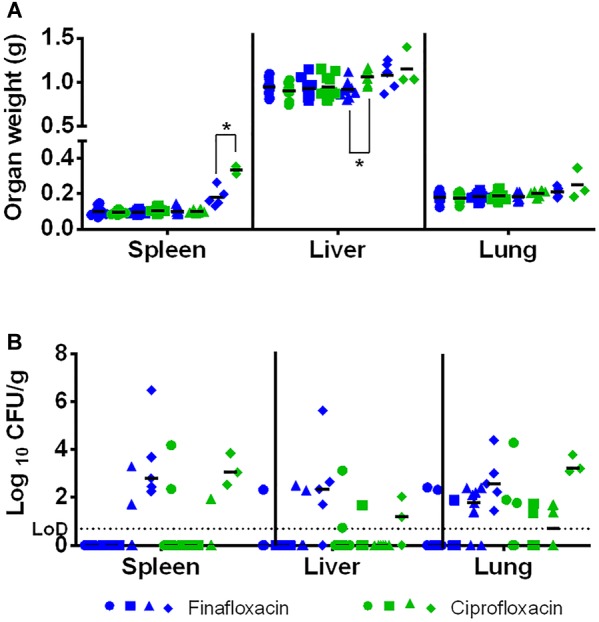
The organ weight and bacterial burden in surviving animals. Mice were challenged with 101 CFU of *F. tularensis* by the intranasal route and treated with finafloxacin (37.5 mg/kg) by the oral route every 8 h or ciprofloxacin (30 mg/kg) by the intraperitoneal route every 12 h. Mice that survived until the end of the study were culled and organs removed and weighed **(A)** and processed for bacterial burden **(B)**. Statistical analysis was performed using the Mood’s Median test with pairwise adjustment to analyze bacterial burden. Circles represent treatment initiated at 24 h, squares 48, triangles 72, and diamonds 96 h post-challenge. ^∗^*p* < 0.05. For the organ weights, the lines represent the means for each group. For the bacterial loads, the lines represent the medians for each group. LOD – limit of detection.

At the end of the study (day 35) the spleens, livers and lungs of all surviving animals were weighed and processed to determine bacterial burden. Mice treated with ciprofloxacin initiated at 72 h and 96 h post-challenge had heavier livers and spleens than mice treated with finafloxacin (both *p* < 0.05) (Figure 8A). There were no further differences in the concentration of bacteria in the organs of mice treated with ciprofloxacin and finafloxacin irrespective of when the therapy was initiated, however, the number of surviving animals colonized at the end of the study did differ (Figure 8B and Table 2). When treatment was initiated at 24 or 48 h post-challenge, more animals treated with ciprofloxacin were colonized at the end of the study when compared to those treated with finafloxacin (40% compared to 10–20%, respectively) (Table 2). When treatment was initiated at 72 or 96 h post-challenge, more animals were colonized at the end of the study (50–78 and 100%, respectively) (Table 2).

**Table 2:** The percentage of surviving mice colonized with *F. tularensis* at the end of the study.

Time of treatment initiation post-challenge (hrs)	Treatment	Proportion of survivors colonized (%)
24	Finafloxacin	20
	Ciprofloxacin	40
48	Finafloxacin	10
	Ciprofloxacin	40
72	Finafloxacin	78
	Ciprofloxacin	50
96	Finafloxacin	100
	Ciprofloxacin	100

*Surviving animals were culled at day 35, the organs harvested and the presence of *F. tularensis* determined.*

## Discussion

Currently, there is no licensed vaccine available which would protect people against either natural exposures or deliberate releases of *F. tularensis*. Antibiotic treatment is typically effective although relapse of disease has been reported in patients treated with ciprofloxacin and doxycycline (Chocarro et al., 2000; Dennis et al., 2001; Pérez-Castrillón et al., 2001; Boisset et al., 2014). This relapse correlates with the development of symptoms and the time taken to initiate therapy (Hepburn and Simpson, 2008). Thus, evaluation of alternative therapies that can be self-administered and that can offer protection even delayed, would offer an obvious advantage to clinicians, negating the use of parenterally administered antibiotics and the associated requirement of both medical facilities and personnel. In addition, therapies offering protection following short regimens would also be of interest, increasing the likelihood of antibiotic regimen completion rates and a reduction in potential side effects.

When evaluated against *F. tularensis* strains SchuS4 and HN63 *in vitro* at pH 7, finafloxacin demonstrated comparable activity to ciprofloxacin (with respect to MIC), but with improved killing ability (as demonstrated by a lower MBC). Finafloxacin demonstrated bactericidal activity against *F. tularensis* strain SchuS4 at pH 7 and bacteriostatic activity at pH 6, although this was very nearly classified as bactericidal. Ciprofloxacin was shown to be bacteriostatic at both pHs. Although finafloxacin did not show superior activity in a time kill assay at pH 6 when compared to pH 7, it was able to rapidly kill bacteria (almost 3 logs in 4 h), a finding previously reported for *B. pseudomallei* (Barnes et al., 2017). This result correlates well with other published work with finafloxacin and other bacterial species *in vitro* (Higgins et al., 2010; Lemaire et al., 2011). It is thought that its bulky structure prevents it from being excluded from bacterial cells by eﬄux pumps, allowing it to remain within the cell retaining its antibacterial activity. pH has also been shown to have an effect on the accumulation of antibiotics within the cell. At acidic pH, an increased accumulation of finafloxacin within eukaryotic cells was demonstrated when compared with ciprofloxacin (Lemaire et al., 2011). Further studies are required to investigate this further.

Seven days of treatment with finafloxacin or ciprofloxacin initiated at 24 h post-challenge provided full protection against an intranasal challenge of *F. tularensis* in mice. Similarly, high levels of protection and clearance in surviving animals were demonstrated when treatment was administered for 3 days, although there was no significant difference between the efficacy of the finafloxacin and ciprofloxacin treatments. This suggests that if administered early, short courses of both antibiotics could have utility. One day of treatment with finafloxacin offered an extension in time to death when compared to ciprofloxacin and resulted in a reduction in bacterial load in the spleen, liver, and lungs. This again may be attributed to finafloxacin having a rapid and improved antibacterial activity in environments where ciprofloxacin is not as effective, demonstrated previously *in vitro* and more recently in urine samples infected with fluoroquinolone resistant pathogens (Lemaire et al., 2011; Vente et al., 2018). No differences were observed between finafloxacin and ciprofloxacin in terms of protection afforded in mice infected with *F. tularensis* or resulting bacterial load when the two antibiotics were administered for 3 or 7 days.

A further study investigated the window of opportunity for initiating treatment. Finafloxacin offered similar levels of protection to ciprofloxacin following initiation of treatment at 24, 48, 72, or 96 h post-challenge. However, stratified analysis of treatment initiated at 72 and 96 h post-challenge, suggested that finafloxacin offered a greater level of protection when compared to ciprofloxacin. Furthermore, when therapy was delayed until 96 h post-challenge, none of the surviving mice treated with finafloxacin relapsed following the cessation of therapy. This data is particularly encouraging considering how severely ill these mice were when therapy was initiated. Mice treated with finafloxacin lost more weight than mice treated with ciprofloxacin, although this dosing regimen of finafloxacin in this mouse strain (BALB/c) has previously shown to result in transient weight loss. Therefore, loss of weight is considered likely to be a side effect of the therapy and not an indicator of the progression of the disease.

At the end of the study there were no major differences with regards to bacterial colonization of the organs of mice treated with finafloxacin or ciprofloxacin. However, mice treated with finafloxacin did have lighter livers and spleens compared to mice treated with ciprofloxacin when therapy was delayed until 72 or 96 h post-challenge, respectively. Acute tularemia in mice following an aerosol challenge is known to result in gross pathological changes in the liver and spleen (Conlan et al., 2003), including an increase in spleen weight (previously demonstrated at 96 h post-challenge in untreated animals, unpublished data). Histological analysis suggested that this weight change is primarily due to cell infiltration (Conlan et al., 2003).

When therapy was delayed until 96 h post-challenge, surviving animals had enlarged spleens (0.18 g and 0.33 g, on average, following finafloxacin and ciprofloxacin therapy, respectively). This, in combination with the bacterial colonization in the spleen at the end of the study, suggests the presence of a chronic infection in these animals. The difference in spleen weight could be due to the different effects of the antibiotics on the bacteria located within the spleen, for example, finafloxacin may have a more rapid effect on colonized bacteria. Alternatively, fluoroquinolones are known to have immunomodulatory activity and finafloxacin may be able to reduce cell infiltration to a greater extent than ciprofloxacin (Dalhoff, 2005). Further histological and immunological analysis would be required to determine the reason for this difference in spleen weight.

The re-emergence of *F. tularensis* infection in mouse models following the cessation of therapy has been well documented, particularly when therapy is delayed (Rotem et al., 2012). The level of colonization during the treatment phase was not determined in this study. For this reason we are unable to confirm whether the surviving animals are colonized due to re-emergence of infection (following cessation of therapy) or because the infection was not adequately controlled initially. Relapse of infection in mice treated with ciprofloxacin (when therapy was delayed until 72 or 96 h post-challenge) at 5 days following cessation of therapy, may suggest that the infection was not adequately controlled. In comparison, relapse following cessation of treatment was only observed in one mouse treated with finafloxacin, suggesting that once administered, this antibiotic is capable of controlling infection. Extending the length of treatment with ciprofloxacin has been shown to improve the level of protection when therapy is delayed (Rotem et al., 2012). Thus, a longer antibiotic dosing regimen may have enabled the infection to have been completely cleared, preventing the relapse of disease, even when therapy was delayed until 96 h post challenge. Increased treatment lengths should be investigated further.

The data generated to date suggests that finafloxacin could be used as an alternative to ciprofloxacin as a prophylaxis or treatment for tularemia. As finafloxacin is available as an oral formulation, this would enable the antibiotic to be self-administered, reducing the burden on medical facilities, which would be particularly important in the event of a deliberate release of *F. tularensis*. Alternatively, an intravenously delivered formulation of finafloxacin has also been developed and could be used as a parenteral treatment in severely ill patients. As finafloxacin has also demonstrated the ability to protect mice against *B. pseudomallei*, it may also have applicability as a broad-spectrum antibiotic for the treatment of infections caused by a wider range of the biothreat agents.

## Data Availability

All datasets generated for this study are included in the manuscript.

## Ethics Statement

The animal studies described in this manuscript were carried out in accordance with the United Kingdom Animal (Scientific Procedures) Act (1986), under a Project License granted by the United Kingdom Home Office.

## Author Contributions

KB, KH, AV, HA, and SH conceived the concept and designed the experiments detailed in the manuscript. KB, KH, and MR conducted the experiments. KB, KH, and TL performed the data analysis. KB, KH, SH, and HA wrote the manuscript. All authors reviewed the draft and approved the manuscript for publication.

## Conflict of Interest Statement

AV is an employee of MerLion Pharmaceuticals. The remaining authors declare that the research was conducted in the absence of any commercial or financial relationships that could be construed as a potential conflict of interest.
